# Targeting SLC7 A11 Ameliorates Ulcerative Colitis by Promoting Efferocytosis Through the ERK1/2 Pathway

**DOI:** 10.1007/s10753-025-02312-6

**Published:** 2025-05-13

**Authors:** Meiyi You, Jichang Li, Xin Wang, Yucun Liu, Shanwen Chen, Pengyuan Wang

**Affiliations:** Department of Gastrointestinal Surgery, Peking University First Hospital, Beijing, 100034 People’s Republic of China

**Keywords:** SLC7 A11, Efferocytosis, Intestinal barrier, Ulcerative colitis

## Abstract

**Objective and design:**

This study investigates the effect and underlying mechanism of targeting SLC7A11 in mitigating dextran sulfate sodium (DSS)-induced intestinal inflammation and injury in colitis.

**Methods:**

We utilized wild-type and SLC7A11^−/+^ mice to assess the inflammatory damage in DSS-induced colitis in vivo. In vitro, colon tissues from patients with ulcerative colitis were analyzed to compare SLC7A11 expression between inflamed and non-inflamed regions. Further mechanistic insights were obtained using Caco-2 cells and bone marrow-derived dendritic cells (BMDCs).

**Results:**

In human colon tissues, SLC7A11 expression was significantly elevated in inflamed regions compared to non-inflamed areas, particularly in dendritic cells. In vivo inhibition of SLC7A11 markedly alleviated DSS-induced colitis symptoms. In vitro, suppressing SLC7A11 restored the integrity of the Caco-2 monolayer intestinal epithelial model. Both knockout and inhibition of SLC7A11 enhanced ERK1/2 phosphorylation and increased efferocytosis in BMDCs.

**Conclusions:**

Targeting SLC7A11 augments dendritic cell efferocytosis and preserves intestinal epithelial barrier function, potentially offering a therapeutic avenue for alleviating ulcerative colitis.

**Supplementary Information:**

The online version contains supplementary material available at 10.1007/s10753-025-02312-6.

## Introduction

Ulcerative colitis (UC) is an idiopathic chronic inflammatory disease of the colonic mucosa. The clinical course is characterized by alternating periods of remission and relapse, with complications such as hemorrhage, obstruction, perforation, and an increased risk of colon cancer. Long-term UC patients face a 10-year cumulative colorectal cancer risk of 2%, escalating to 18% over 30 years [1]. The global incidence of UC has risen, with developed countries experiencing high rates and developing nations observing significant increases [2]. The etiology of ulcerative colitis is unknown, and its susceptibility may be related to genetic factors, environmental factors, and previous gastrointestinal infections [3]. Current treatments-mesalazine, corticosteroids, immunosuppressants, and TNF-α monoclonal antibodies-fail to induce remission in over half of patients, with initial responders often relapsing [4]. Restorative colectomy is still required for patients who are refractory to medical therapy or who have colitis-related colorectal dysplasia or cancer [5]. These challenges significantly impact patients'quality of life, underscoring the need for novel therapeutic targets to mitigate inflammation and preserve intestinal barrier integrity.

Efferocytosis, the clearance of apoptotic cells by phagocytes, is vital for tissue homeostasis. Deficiencies in this process contribute to chronic inflammatory diseases [6]. Cells undergo apoptosis and release a number of factors known as “find me” signals, which recruit phagocytes to the site of death [7]. Once phagocytes arrive, they must distinguish between dead and live cells. As part of the apoptosis procedure, cells are exposed to phosphatidylserine, which acts as a “eat-me” signal for phagocytes [8]. Phagocytic cells recognize phosphatidylserine through receptors such as MerTK, TIM-4 and αvβ3/5 [9], inducing phagocytosis of apoptotic cells. Defects in the burial mechanism caused by the ablation of the phosphatidylserine receptor CD300f have been implicated in inflammatory bowel syndrome and colitis, as well as the progression of colon cancer [12]. Efferocytosis also has an important immune function, often performed in an “immunosilencing” manner, characterized by the secretion of the hallmark immunosuppressive cytokines interleukin-10 (IL-10) and transforming growth factor β (TGF-β) [13]. Recent studies have shown that abnormal efferocytosis is involved in the pathogenesis of UC [14]. The occurrence and development of UC is associated with delayed neutrophil apoptosis and neutrophil-mediated damage [15]. Enhancing efferocytosis may ameliorate barrier dysfunction in UC, as demonstrated in murine models where dendritic and epithelial cells mediated disease control through apoptotic cell clearance [16]. Intestinal epithelial cells undergo continuous renewal, necessitating efficient apoptotic cell removal to prevent inflammation [17]. While epithelial cells possess phagocytic capabilities, dendritic cells and macrophages are primary efferocytosis in the gut [18].

The solute carrier (SLC) family is the second largest gene family in the human genome (after G protein-coupled receptors) and mediate the transport of metabolites and solutes across cell membranes [19]. SLC7 A11 belongs to the SLC7 family and is an amino acid transporter. It is a non-Na^+^-dependent electroneutral exchanger of extracellular cystine and intracellular glutamate [20]. Efferocytosis contributes to the recovery of inflammation, but inflammatory diseases occur when phagocytic cells are unable to process the biomass and metabolic load of phagocytosed apoptotic cells [21]. In recent studies, SLC7 A11 has been shown to inhibit the efferocytosis of phagocytic cells, such as dendritic cells. Knockdown of SLC7 A11 expression can enhance the efferocytosis of dendritic cells and promote wound healing [22]. Efferocytosis intersects with immune and metabolic pathways, influencing immune responses through metabolic modulation [23]. Given the druggability of SLC transporters and their impact on immune cell function, understanding the mechanisms by which SLC7 A11 regulates efferocytosis is imperative [24]. We found that targeting SLC7 A11 was able to enhance efferocytosis, reduce inflammation, and restore intestinal barrier function in patients with ulcerative colitis to alleviate ulcerative colitis. Elucidating this relationship may reveal new insights into the pathogenesis of ulcerative colitis and provide new avenues for treatment.

## Materials and Methods

### 1 A4

To prepare the monoclonal antibody, we used multiple transmembrane protein SLC7 A11 overexpression in 293 F cells as an immunogen and immunized BALB/c mice with subcutaneous multi-point injection at a dose of 1 × 10^6^ cells/mouse. After multiple doses, we collect mouse serum for flow cytometry testing to ensure that the immune response has reached an adequate level. Subsequently, we collected splenocytes from immunized mice and fused them with myeloma cells to form hybridoma cells. Successfully confluent hybridoma cells are screened by selective medium and then restricted dilution to obtain monoclonal hybridoma cells. Each cloned cell was cultured independently, and the antibodies in the culture were finally collected for flow cytometry titer testing to select the monoclonal cells with the highest titer. After selecting the monoclonal cells with the highest titers, we performed expansion and antibody production. After purification and characterization, the monoclonal antibodies (1 A4) obtained are used in subsequent experiments.

### Animals and Models

The SLC7 A11 knockdown mouse was created by Gempharmatech using a CRISPR/Cas9-mediated genome-editing system. SLC7 A11 gene has 5 transcripts. According to the structure of SLC7 A11 gene, exon2 of SLC7 A11-201(ENSMUST00000029297.6) transcript is recommended as the knockout region. The region contains 127 bp coding sequence. Knock out the region will result in disruption of protein function. CRISPR/Cas9 system were microinjected into the fertilized eggs of C57BL/6 JGpt mice. Fertilized eggs were transplanted to obtain positive F0 mice which were confirmed by PCR and sequencing. A stable F1 generation mouse model was obtained by mating positive F0 generation mice with C57BL/6 JGpt mice. For animal experiments with SLC7 A11^−/+^ mice, littermate controls with normal SLC7 A11 expression were used as WT animals. Animals were randomly allocated to experimental groups, and all SLC7 A11^−/+^ mice used in this study were male mice of matched age. The mice were maintained in specific pathogen-free facilities with a standard 12 h light/dark schedule and provided standard chow and water ad libitum at the Department of Laboratory Animal Science of Peking University. The mouse model of colitis was constructed: DSS (Yeasen) was free to drink water of 3 percent for seven days. Sulfasalazine (SAS) is a traditional treatment for ulcerative colitis. Grouping includes: (1) Control; (2) DSS (WT) + PBS (10 μl/kg; Intraperitoneal injection; Day:0,2,4,6); (3) DSS (WT) + 1 A4 (10 mg/kg; Intraperitoneal injection; Day:0,2,4,6); (4) DSS (SLC7 A11^−/+^) + PBS (10 μl/kg; Intraperitoneal injection; Day:0,2,4,6); (5) DSS (SLC7 A11^−/+^) + 1 A4 (10 mg/kg; Intraperitoneal injection; Day:0,2,4,6); (6) DSS (WT) + SAS (10 mg/kg; Intragastric injection; Day:0–6).

### Disease Activity Index (DAI)

Scoring system for calculating a disease activity index based on weight loss, stool consistency and the degree of intestinal bleeding, as shown in Table 1 [26].

**Table 1:** Disease Activity Index

Score	Weight loss	Stool consistency	Blood
0	None	Normal	Negative hemocult
1	1–5%	Soft but still formed	Negative hemocult
2	6–10%	Soft	Positive hemocult
3	11–18%	Very soft; wet	Blood traces in stool visible
4	> 18%	Watery diarrhea	Gross rectal bleeding

### Histological Assessment of Colon Epithelium

The proximal colons of different groups of mice were taken and embedded with paraffin. We prepared sections and used Hema-toxylin and Eosin (HE) to stain them. We obtained the images using Zeiss imaging optical microscopes with magnifications of 40 ×, 100 ×, and 400 ×. We collected at least 6 images for each slice. The extent of histopathological alterations was assessed by 2 pathologists who were unaware of grouping and grading based on a scoring method as previously mentioned.Three independent scoring parameters were determined: Severity of inflammation (score 0 to 3: none, mild, moderate, severe), depth of injury (score 0 to 3: none, mucosa, mucosa and submucosa, transmural), and crypt damage (0 to 4: none, basal 1/3 damaged, basal damaged, superficial epithelium intact, invasion of the entire crypt and loss of epithelium). The score for each parameter is multiplied by a factor that reflects the percentage of tissue involvement (× 1, 0%−25%; × 2, 26%−50%; × 3, 51%−75%; × 4, 76%−100%) and add. The maximum possible score is 40 [27].

### Western Blotting (WB)

The total protein of tissue and cells was extracted with RIPA. BCA method (Lablead) was used to detect the protein concentration, and the extracts containing same amount of proteins (30 μg) were cataphoresed in 4%−12% polyacrylamidegel. Isolated protein was transferred onto the PVDF membrane. The membrane is blocked with NcmBlot blocking buffer for 20 min at room temperature. Rabbit anti-XCT monoclonal antibody (1:1000 dilution; Abcam), mouse anti-GAPDH monoclonal antibody (1:1000 dilution; CST), rabbit anti-ZO-1 monoclonal antibody (1:1000 dilution; Thermo Fisher), mouse anti-Occludin monoclonal antibody (1:1000 dilution; Thermo Fisher), rabbit anti-MLCK monoclonal antibody (1:1000 dilution; CST), rabbit anti-MLC2 monoclonal antibody (1:1000 dilution; CST), rabbit anti-P-MLC2 monoclonal antibody (1:1000 dilution; CST), rabbit anti-ERK1/2 monoclonal antibody (1:1000 dilution; CST), rabbit anti-P-ERK1/2 monoclonal antibody (1:1000 dilution; CST), rabbit anti-AXL monoclonal antibody (1:1000 dilution; Abcam)were used to incubate the membrane for the night at 4℃. Then we used the secondary antibody to incubate the membrane for 1 h at room temperature, and used ECL detection reagent to perform blots (Lablead).

### Immunohistochemistry

Sections (4 μm) of paraffin-embedded formalin-fixed tissue were deparaffinized with xylene and rehydrated by fractional concentrations of ethanol. Microwave antigen retrieval of sections was with 0.1 M EDTA buffer (pH 9.0) at 95℃ for 15 min. Sections were blocked with goat serum (ZSGB-BIO) and incubated with endogenous peroxidase for 30 min at room temperature. Then, combined with rabbit anti-MPO monoclonal antibody (1:100 dilution; Proteintech), rabbit anti-XCT monoclonal antibody (1:100 dilution; Proteintech) at 4℃ overnight. After incubation with DAB for 1 min, counterstained with hematoxylin. Estimation of the number of MPO-positive and XCT-positive epithelial cells was performed independently by 2 pathologists without knowledge of the clinicopathological parameters. For each slide, at least 3 microscopic fields were randomly chosen, and the intensity of staining was then calculated for each group.

### Immunofluorescence

Rinsed the Caco-2 monolayer with PBS and fixed overnight with 100% methanol at −20℃, followed by 100% acetone at −20℃ for 1 min. Then blocked with 1% bovine serum albumin for 1 h at room temperature. Subsequently, the filter was incubated with 6 μg/ml mouse monoclonal anti-ZO-1 antibody (Thermo Fisher) and 4 μg/ml rabbit monoclonal anti-occludin antibody (Thermo Fisher) overnight at 4℃. Sections (4 μm) of paraffin-embedded formalin-fixed tissue were deparaffinized with xylene and rehydrated by fractional concentrations of ethanol. Microwave antigen retrieval of sections was with 0.1 M EDTA buffer (pH 9.0) at 95℃ for 15 min. Sections were blocked with goat serum (ZSGB-BIO) and incubated with endogenous peroxidase for 30 min at room temperature. Then, combined with rabbit anti-CD11c antibody (CST 1:200) and mouse anti-XCT antibody (Proteintech 1:200) at 4℃ overnight. After washing with PBS, the filter and sections were incubated with goat anti-rabbit IgG conjugated to Alexa Fluor 488 (1:300; Thermo Fisher) and goat anti-mouse IgG conjugated to Alexa Fluor 647 (1:200; Thermo Fisher) for 1 h at room temperature. After washing with PBS, mounted the slides with Prolong Gold Antifade Reagent (Thermo Fisher) and stored at 4℃ protected from light until analysis. The fluorescence was visualized on an LSM710 confocal microscope (Carl Zeiss).

### Flow Cytometry

Immunophenotyping of human colon tissue performed on single-cell suspensions. Cells were stained with the following anti-human, monoclonal, fluorochrome-linked antibodies: BD Horizon™ BV421 Mouse Anti-Human CD45; BD OptiBuild™ BV605 Mouse Anti-Human CD8; BD Horizon™ BUV395 Mouse Anti-Human CD3; BD Horizon™ BUV395 Mouse Anti-Human CD4; BD Horizon™ BUV737 Mouse Anti-Human CD19; BD Pharmingen™ PE-Cy™7 Mouse Anti-Human CD56 (NCAM-1); BD Pharmingen™ Alexa Fluor® 700 Mouse anti-Human CD11b; BD Horizon™ BV510 Mouse Anti-Human CD14; BD Horizon™ BB515 Mouse Anti-Human CD11c; APC/Cyanine7 anti-human CD326 (Ep-CAM) Antibody; BD Pharmingen™ 7-AAD;PE-SLC7 A11; BD Horizon™ AF647 anti-human FOXP3. Measurements were performed on a BD LSR Fortessa cytometer. Analysis was used with FlowJo10 software.

### Efferocytosis Assays

For induction of apoptosis, human Jurkat T cells were stained with pHrodo Green STP Ester (P35369, Thermo Fisher), resuspended in RPMI with 5% fetal calf serum, treated with staurosporine (0.5 μg/ml) for 3 h and incubated for 4 h at 37 ℃ with 5% CO_2_. BMDCs were incubated with apoptotic targets in a 1:5 phagocyte: target ratio for 2 h. Cells were stained with the following anti-mouse, monoclonal, fluorochrome-linked antibodies: 7-AAD Viability Staining Solution (Biolegend), Brilliant Violet 421™ anti-mouse CD11c (Biolegend).

### Terminal Deoxynucleotidyl Transferase (TdT)-Mediated dUTP Nick End Labeling (TUNEL)

TUNEL assey used One-step TUNEL In Situ Apoptosis Kit (Green, FITC, Elabscience). The tissue sections were deparaffinized and hydrated and the cells were permeabilized by incubation at 37℃ for 20 min with proteinase K. Washed the sections with PBS to remove any residual fixative or detergent. Equilibrated with TdT Equilibration Buffer at 37℃ for 10–30 min, ready for use in the TUNEL reaction. Incubated with TUNEL reaction mixture containing TdT enzyme and fluorophore-labeled FITC-dUTP for 60 min. After washing with PBS, mounted the slides with Prolong Gold Antifade Reagent (Thermo Fisher) and stored at 4℃ protected from light until analysis.

### Transepithelial Electrical Resistance (TEER) Measurements

In a previous study [28], a 12-well Transwell setup was employed for our experiment. To begin, Caco-2 cells were plated within the apical compartment, which was immersed in 1.5 ml of growth medium contained in the basal chamber. The transepithelial electrical resistance (TEER) was then quantified using an epithelial volt-ohm meter provided by World Precision Instruments. Once the Caco-2 cell layers had fully integrated, typically after about three weeks, exhibiting TEER readings between 400 to 550 Ω·cm^2^. We made adjustment to the TEER values to account for any background resistance contributed by the membrane itself. These corrected TEER values were expressed in units of Ω·cm^2^. We continued to monitor electrical resistance until we obtained three successive measurements that were consistent with one another, indicating stability in the epithelial barrier's resistance.

### FITC-Dextran 4(FD-4) Flux Measurements

On Transwell filters, Caco-2 cells were cultivated to form monolayers and subsequently treated as previously described [28]. Following this, the cells underwent a 2-h incubation in Hank’s balanced salt solution, which was supplemented with a 1 mg/ml FD-4 solution. To evaluate the FD-4 flux, a 100 μl sample was extracted from the basolateral chamber. The fluorescent signal’s intensity was then quantified with a Synergy H2 microplate reader (Bio Tek Instruments), utilizing an excitation wavelength of 492 nm and an emission wavelength of 520 nm. The FD-4 concentration was ascertained by comparing it with standard curves that were established through the serial dilution of FD-4.

### Cell Culture

We acquired the human colonic cell lines Caco-2 from the American Type Culture Collection. Caco-2 was cultured in DMEM High Glucose (Gibco), supplemented with 10% v/v fetal bovine serum (Sigma), penicillin (50 U/ml), streptomycin (50 U/ml). Incubation was in an environment of 37℃ and 5% CO_2_. For the in vitro experiments, 10 ng/ml TNF-α/IFN-γ (Peprotech) was added into the medium for 48 h to induce the cell damage with or without 1 A4 (200 μg/ml). BMDCs were generated from mice by culturing bone marrow progenitors for 7 days in GM-CSF (Peprotech)-supplemented and IL-4 (Peprotech)-supplemented medium.

### scRNA-seq Data Analysis

The Data generated from colonic mucosa in the ulcerative colitis subjects included can be accessed on NIH GEO (https://www.ncbi.nlm.nih.gov/geo/) with the accession number GSE9452.

### Statistical Analysis

Statistical significance was determined using GraphPad Prism 9, using unpaired two-tailed T test or Multiple T test. *P < 0.05, **P < 0.01, ***P < 0.001; #P < 0.05, ##P < 0.01, ###P < 0.001.

## Results

### The Expression Level of SLC7 A11 was Significantly Increased in Inflamed Colon Epithelial Tissues Collected from UC Patients

**Fig.1:**
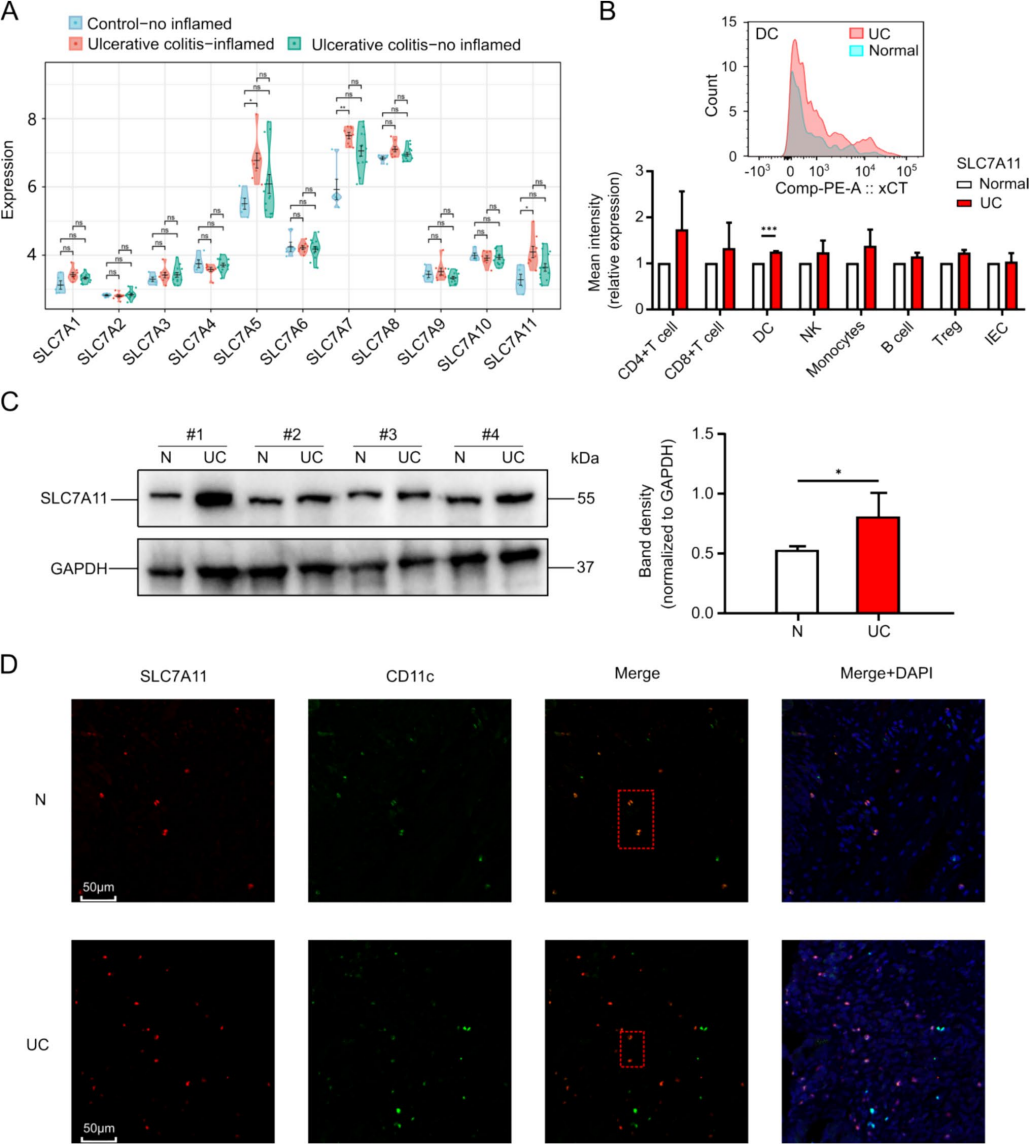
The expression level of SLC7 A11 was significantly increased in inflamed colon epithelial tissues collected from UC patients**. A** RNA sequencing results showed that the expression level of SLC7 A11 was in Control − no inflamed, Ulcerative colitis − inflamed and Ulcerative colitis − no inflamed. **B** The above figure was a representative plot of the SLC7 A11 expression in DC, and the bottom figure was relative amount of fluorescence intensity of SLC7 A11 counted by flow cytometry in inflamed colonic epithelial tissue and normal colonic epithelial tissue of UC patients(n = 3).**C** Protein levels were analyzed by paired western blotting SLC7 A11 in inflamed colonic epithelial tissue and normal colonic epithelial tissue in UC patients(n = 4). **D** Immunofluorescence showed that the localization and expression of SLC7 A11 and CD11c in the inflamed colonic epithelial tissue and normal colonic epithelial tissue of UC patients. (* p < 0.05, ** p < 0.01, *** p < 0.005)

Studies have demonstrated a strong association between the SLC7 family and inflammatory immune response [29]. RNA sequencing data revealed that expressions of SLC7 A5, SLC7 A7, and SLC7 A11 in colon tissues of ulcerative colitis (UC) patients were significantly elevated compared to non-inflamed individuals (Fig. 1A). The core functions of SLC7 A5/SLC7 A7 (amino acid metabolism and lysosomal transport) [30, 31] are weakly correlated with key pathological links such as intestinal inflammation, barrier damage or microbiota interaction in IBD. It has been reported in the literature that SLC7 A11 is related to the role of efferocytosis [22], and efferocytosis is related to the occurrence and development of inflammation [6]. Notably, the link between SLC7 A11 and UC has not been extensively studied. Flow cytometry analysis of colon tissues from UC patients indicated increased SLC7 A11 expression in inflamed regions across various immune cells(Fig.S1A), with a pronounced elevation in dendritic cells (DCs) (Fig. 1B). Additionally, the number of DCs in inflamed tissues was elevated(Fig.S2 A), suggesting a potential relationship between inflammation and altered cellular phagocytic activity. Western blot analysis comparing inflamed and paired non-inflamed colon tissues from UC patients confirmed a significant upregulation of SLC7 A11 in inflamed epithelial tissues (Fig. 1C). Immunofluorescence indicated that SLC7 A11 colocalized with CD11c (Fig. 1D), which is in accordance with previous results [20]. Collectively, these findings indicate that SLC7 A11 expression is markedly increased in inflamed colon tissues of UC patients, particularly within dendritic cells, suggesting a potential role of SLC7 A11 in the pathogenesis of UC.

### SLC7 A11 Monoclonal Antibody (1 A4) Ameliorated Survival, Weight Loss, DAI, Colon Length, and Preserved the Intestinal Barrier in a DSS-Induced Mouse Colitis Model

**Fig. 2:**
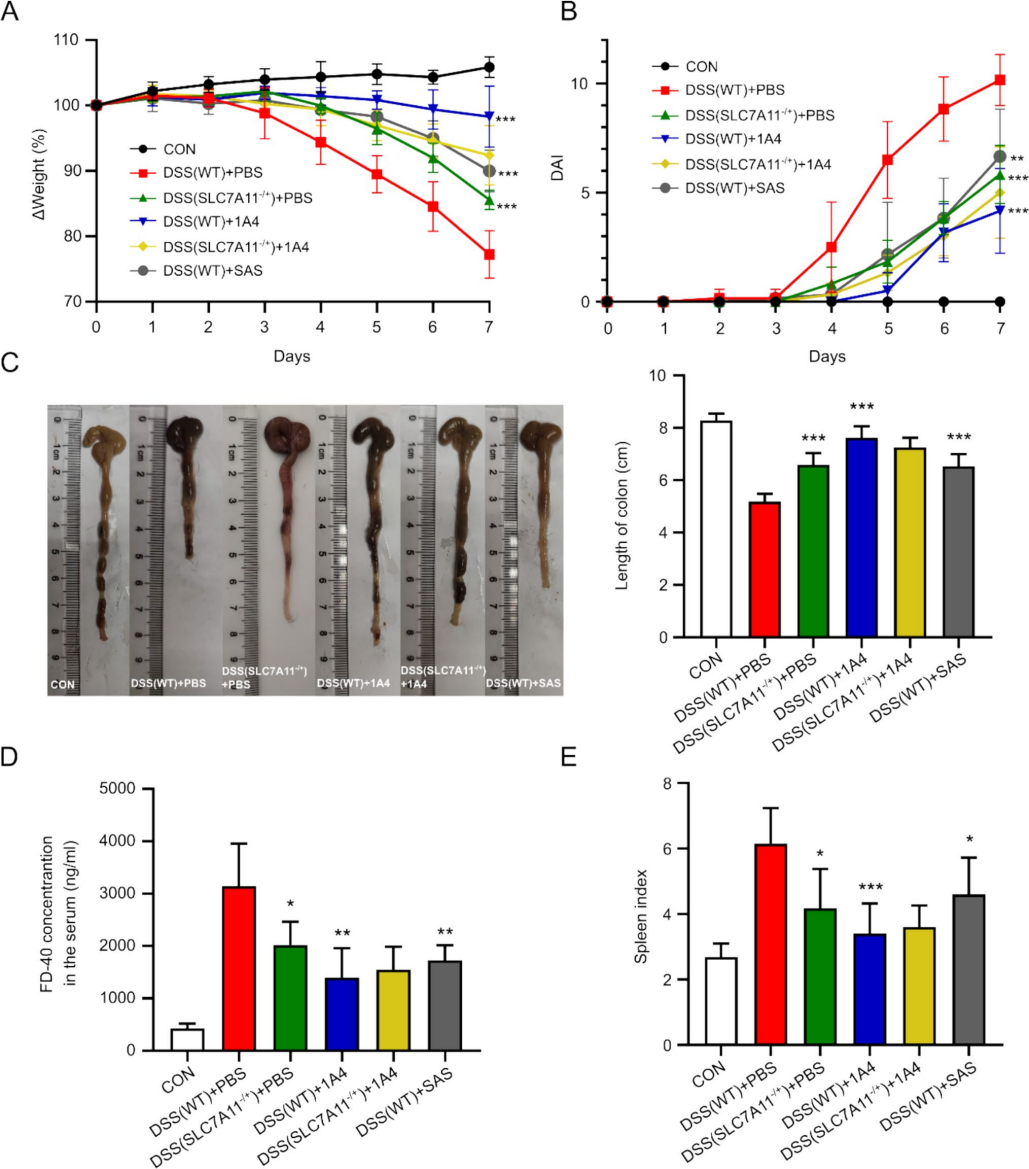
SLC7 A11 monoclonal antibody (1 A4) Ameliorated Survival, Weight Loss, DAI, Colon Length, and preserved the intestinal barrier in a DSS-Induced Mouse Colitis Model**. A** Change in body weight of WT and SLC7 A11^−/+^ mice in each group over 7 days(n = 6). **B** Changes in DAI of WT and SLC7 A11^−/+^ mice in each group within 7 days(n = 6). **C** Colon length of WT and SLC7 A11^−/+^ mice intestines in each group at day 7(n = 6). **D** FD-4 flux in the intestines of WT and SLC7 A11^−/+^ mice on day 7(n = 6). **E** Spleen index on day 7 of WT and SLC7 A11^−/+^ mouse. Spleen index: (Weight of spleen/Weight of mouse) × 10(n = 6). (* p < 0.05, ** p < 0.01, *** p < 0.005 vs DSS(WT) + PBS; # p < 0.05, ## p < 0.01, ### p < 0.005 vs DSS(SLC7 A11.^−/+^) + PBS)

WB validated knockout efficiency in SLC7 A11^−/+^ mice (Fig.S2B). Compared to the other groups, the body weight of mice in the DSS + PBS group decreased significantly after 7 days of DSS treatment (Fig. 2A). 1 A4 treatment or SLC7 A11^−/+^ mice alleviated DSS-induced weight loss. However, there was no significant difference between the DSS (SLC7 A11^−/+^) + PBS and DSS (SLC7 A11^−/+^) + 1 A4 groups. The DAI results (Fig. 2B) showed that the mice in the DSS (WT) + PBS group had the highest scores, which was consistent with the results of weight loss. The DSS (WT) + 1 A4 group had the lowest score. DSS resulted in a shortening of colon length in WT mice, while 1 A4 treatment increased colon length in WT mice (Fig. 2C). Serum FD-4 was significantly increased in the DSS (WT) + PBS group compared to the 1 A4 group and DSS (SLC7 A11^−/+^) + PBS group (Fig. 2D). The spleen index, calculated as the spleen weight relative to body weight, serves as an indicator of immune function [32]. In this study, the spleen index was significantly elevated in the DSS(WT) + PBS group compared to both the 1 A4-treated group and the DSS (SLC7 A11^−/+^) + PBS group. This suggests that DSS-induced colitis leads to splenic enlargement, which is mitigated by 1 A4 treatment and SLC7 A11 heterozygosity(Fig. 2E). These findings indicate that targeting SLC7 A11 effectively protects the intestinal barrier and reduces inflammation in DSS-induced colitis models, demonstrating superior performance compared to sulfasalazine.

### 1 A4 Ameliorated the Histological Damage and Apoptosis in Mouse Models with Colitis

**Fig. 3:**
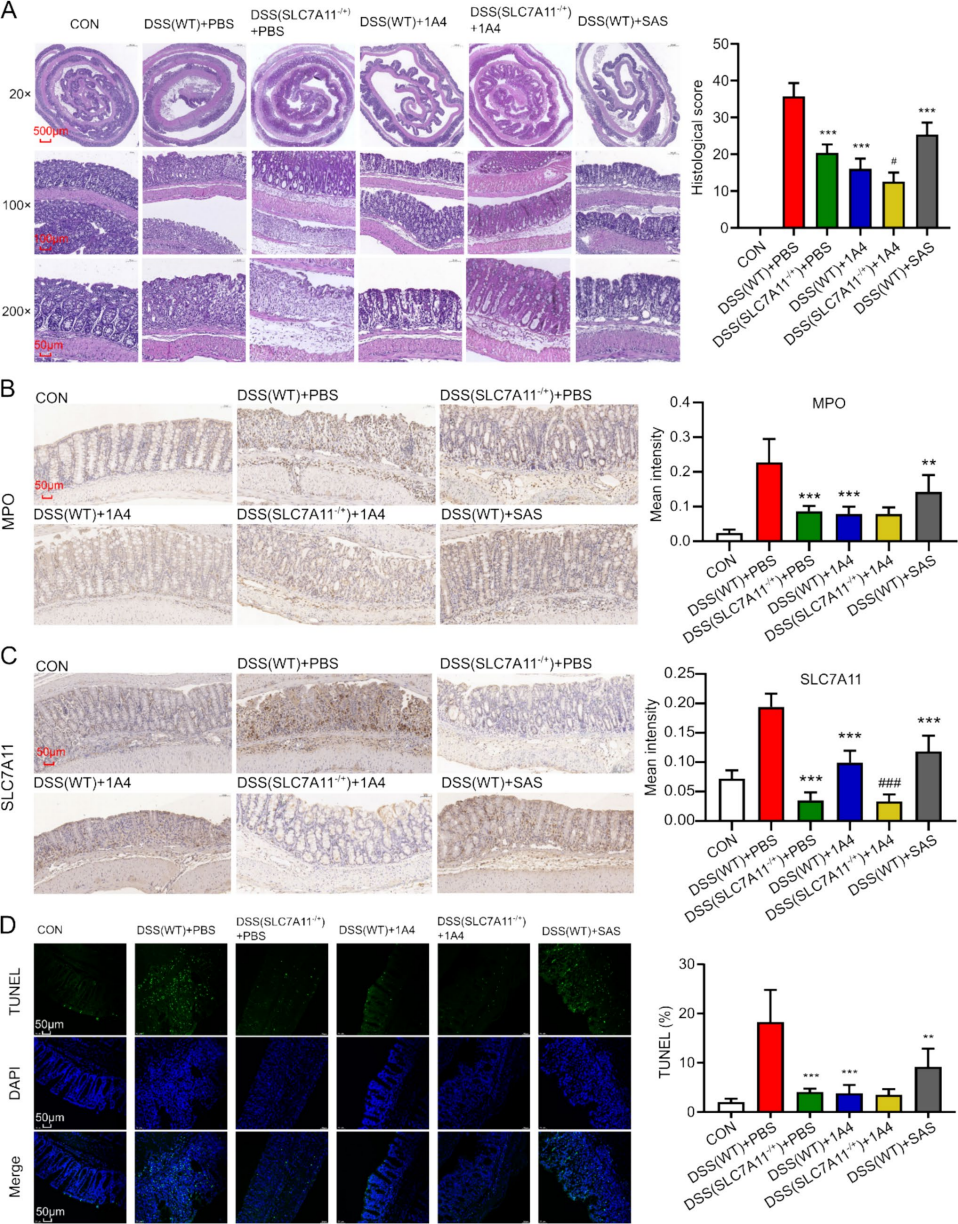
1 A4 ameliorated the histological damage and apoptosis in mouse models with colitis

After 7 days of DSS treatment, HE staining was used to score the intestinal epithelial tissue damage in each group (Fig. 3A). The results showed that DSS (WT) + 1 A4 group and DSS (SLC7 A11^−/+^) + PBS group attenuated the histological damage of the intestinal epithelial of mice compared with DSS (WT) + PBS group. MPO was a peroxidase enzyme that was mainly found in neutrophils and monocytes and involved in the process of antimicrobial and oxidative stress. By immunohistochemistry scoring, it was seen that MPO expression in group DSS (WT) + 1 A4 was significantly lower than in group DSS (WT) + PBS and (Fig. 3B). There was no significant difference in expression intensity between the DSS (SLC7 A11^−/+^) + PBS and DSS (SLC7 A11^−/+^) + 1 A4 groups. The expression level of SLC7 A11 was lowest in the DSS (SLC7 A11^−/+^) + PBS and DSS(SLC7 A11^−/+^) + 1 A4 groups (Fig. 3C), which may be due to the fact that SLC7 A11 was knocked out and 1 A4 no longer reduced the expression level of SLC7 A11 after gene knockout. The expression level of SLC7 A11 was also reduced in the DSS (WT) + 1 A4 and DSS(WT) + SAS groups which was consistent with the trend of MPO, suggesting that the expression level of SLC7 A11 may be consistent with the degree of inflammation. 1 A4 and SAS attenuated colonic inflammation and reduced SLC7 A11 expression in mice. After 1 A4 treatment, there were fewer apoptotic cells in colon tissue (Fig. 3D). These results suggested that 1 A4 was effective in alleviating inflammation and colonic injury and reduced apoptosis positivity in DSS-induced colitis models.

**A** Representative image of HE staining of WT and SLC7 A11^−/+^ mice colon tissue with or without 1 A4 after DSS administration at day 7 (20 ×, 100 ×, and 200 ×) and histological lesion scores. **B** Representative images of immunohistochemistry of MPO and mean expression intensity. **C** Representative images of immunohistochemistry of SLC7 A11 and mean expression intensity. **D** Representative images of immunofluorescence of apoptotic cell and positivity rate (TUNEL/DAPI). (* p < 0.05, ** p < 0.01, *** p < 0.005 vs DSS(WT) + PBS; # p < 0.05, ## p < 0.01, ### p < 0.005 vs DSS(SLC7 A11^−/+^) + PBS).

### 1 A4 relieved TNF-α/IFN-γ-Induced Caco-2 Monolayer Barrier Injury

**Fig. 4:**
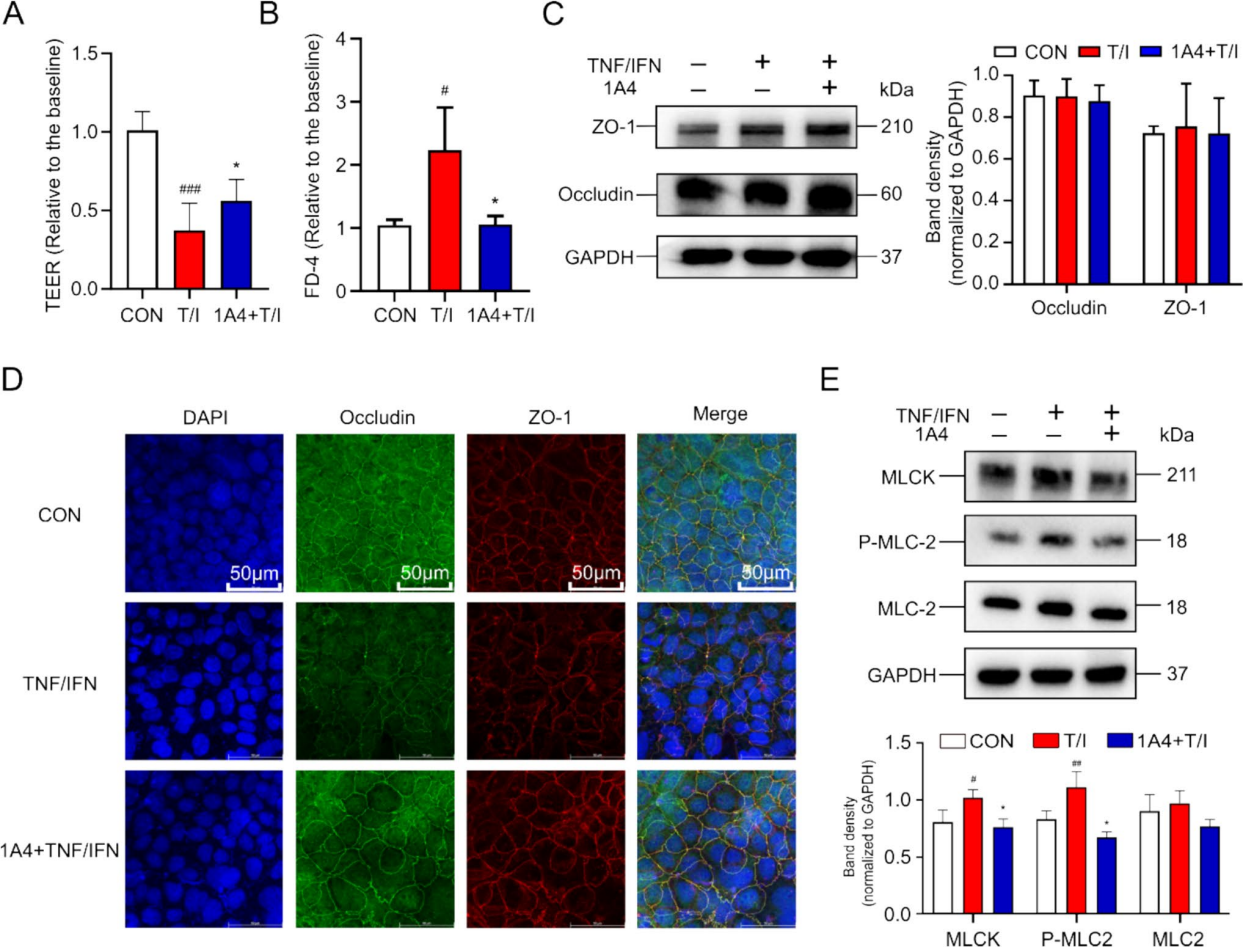
1 A4 relieved TNF-α/IFN-γ-induced Caco-2 monolayer barrier injury. **A** TEER at 48 h in comparation with baseline after treatment with1 A4 with or without TNF/IFN. **B** FD-4 flux at 48 h in comparation with baseline after different treatments as described in A. **C** The expression of tight junction proteins ZO-1 and Occludin in Caco-2 monolayers from groups with different treatments. The total proteins were collected 48 h after baseline, and western blotting assays were performed. **D** Immunofluorescent staining of ZO-1 and Occludin in Caco-2 cell monolayers grown on filters. **E** The expression of MLCK protein and the phosphorylation level of MLC were analyzed by western blotting. (* p < 0.05, ** p < 0.01, *** p < 0.005)

1 A4 significantly alleviated TNF-α/IFN-γ-induced barrier function damage and alleviated the reduction in TEER values (Fig. 4A). The results of the FD-4 flux assay were consistent with the TEER measurements (Fig. 4B). However, there was no significant difference in the expression of tight junction proteins, including ZO-1 and Occludin (Fig. 4C). However, TNF/IFN-induced localization of ZO-1 and Occludin in the Caco-2 monolayer was significantly altered, and cell membrane immunofluorescence showed a curved staining pattern for these proteins. Targeting SLC7 A11 mitigated these TNF/IFN-induced changes, reducing the degree of irregularity in fluorescent staining for both proteins (Fig. 4D). The disruption of tight junction protein localization, induced by TNF and IFN, was due to the contraction of the F-actin ring that encircled the tight junction of the intestinal epithelial cell membrane. This contraction of F-actin was a highly dynamic process, with the MLCK-P-MLC signaling pathway having been identified in previous studies as a key regulator of F-actin within intestinal epithelial cells [33]. Therefore, we further investigated the activation of the MLCK-P-MLC signaling pathway in the Caco-2 monolayer membrane. The results showed that inhibition SLC7 A11 not only induced a significant decrease in MLCK expression, but also a decrease in TNF/IFN-induced levels of MLC-2 phosphorylation (Fig. 4E). These resutls indicated that targeting SLC7 A11 preserves the monolayer barrier function through inhibiting MLC-MLCK signaling pathway.

### 1 A4 Enhanced the Efferocytosis of BMDC by ERK1/2 Pathway

**Fig. 5:**
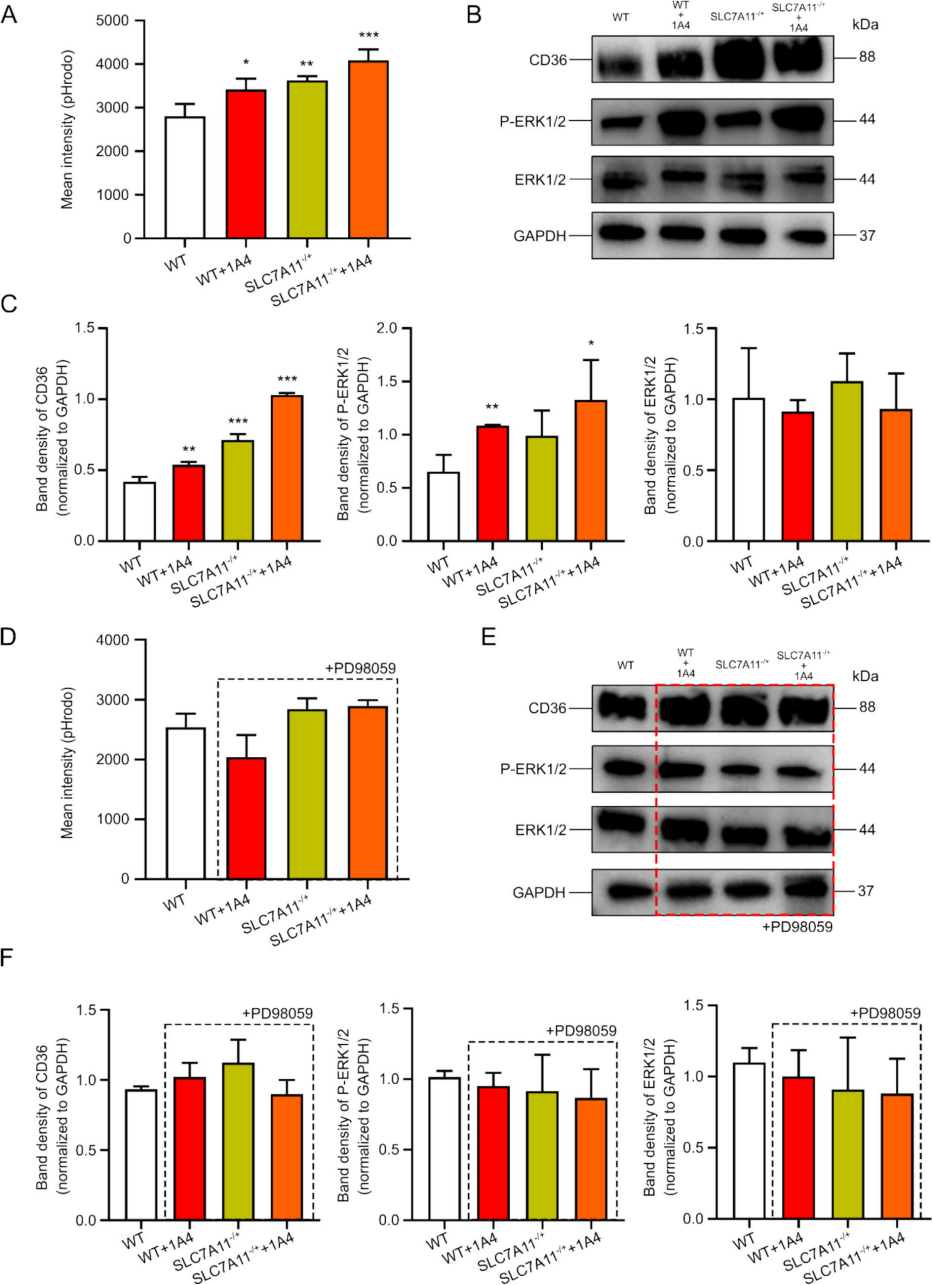
**1 A4 enhanced the efferocytosis of BMDC by ERK1/2 pathway. A** Detection of cell efferocytosis with pHrodo fluorescence intensity of WT and SLC7 A11.^−/+^ BMDCs with or without 24 h treatment of 1 A4. **B-C** The expression of ERK protein, CD36 protein and the phosphorylation level of ERK were analyzed by western blotting. **D** 10 μM PD98059 was added to the other groups except WT BMDC for 22 h. Detection the intensity of efferocytosis as described in A. **E–F** The expression of ERK protein, CD36 protein and the phosphorylation level of ERK were analyzed by western blotting with or without 22 h treatment of PD98059. (* p < 0.05, ** p < 0.01, *** p < 0.005)

We inhibited SLC7 A11 expression by 1 A4 and extracted primary cells from SLC7 A11^−/+^ mouse bone marrow to observe the effect of targeting SLC7 A11 on efferocytosis of BMDC. The addition of 1 A4 to BMDCs with WT or SLC7 A11^−/+^ significantly increased the efferocytosis intensity of BMDCs (Fig. 5A). The efferocytosis intensity of the SLC7 A11^−/+^ BMDC itself was also higher than that of the WT BMDC. Previous studies had shown that the ERK1/2 pathway is an important pathway for efferocytosis [34]. The expression of ERK1/2 after BMDC efferocytosis of apoptotic cells was detected by WB, and the results showed that the phosphorylation level of ERK1/2 increased significantly after inhibition of SLC7 A11(Fig. 5B and 5 C). The expression level of CD36, a molecular marker of efferocytosis, was also significantly increased. After the addition of ERK inhibitor PD98059, the promoting effect of inhibition SLC7 A11 on the efferocytosis of BMDC disappeared (Fig. 5D, 5E, 5 F). These results suggested that targeting SLC7 A11 promoted efferocytosis of dendritic cells through the ERK1/2 pathway.

## Discussion

SLC7 A11 as a solute transporter, is gaining attention for its role in inflammation related diseases [29]. However, the role of SLC7 A11 in the pathogenesis of UC and its underlying mechanisms remain to be fully elucidated. Apoptosis of inflammatory cells and efferocytosis of phagocytic cells play an important role in promoting inflammatory remission, and intestinal monocyte-macrophage populations can secrete mediators that promote repair [37]. Studies [21] have shown that inhibition of SLC7 A11 can promote cytogenesis by increasing glycogenolysis and increasing the rate of aerobic glycolysis [22], and SLC7 A11 blockers can improve wound healing kinetics when used in combination with apoptotic cells. Based on these results, we set out to investigate whether targeting SLC7 A11 can alleviate ulcerative colitis by promoting efferocytosis.

In this study, we compared SLC7 A11 expression in inflamed and non-inflamed colonic epithelial tissues from UC patients. Protein-level analysis revealed a significant upregulation of SLC7 A11 in inflamed epithelial tissues. Flow cytometry indicated increased SLC7 A11 expression in immune and intestinal epithelial cells within inflamed regions, particularly in dendritic cells, with notable colocalization of SLC7 A11 and CD11c. These findings suggest that targeting SLC7 A11 may enhance efferocytosis in DCs, potentially alleviating UC symptoms.

In this study, DSS induced WT and SLC7 A11^−/+^ colitis models in mice. The results showed that after ad libitum drinking DSS, the body weight and clinical scores of mice in the intraperitoneal PBS group significantly reduced, which was faster than that of the mice intraperitoneally injected with 1 A4 and mice of SLC7 A11^−/+^, with significantly increased intestinal permeability, more significant colon shortening, and higher spleen index. In addition, histological evaluation of the colon in mice showed a reduction in the degree of inflammation of the colon tissue after targeting SLC7 A11 therapy. Immunohistochemistry showed a decrease in MPO levels in colon tissue. The results of TUNEL staining showed that the proportion of apoptotic cells in the colon of mice injected intraperitoneally with 1 A4 and SLC7 A11^−/+^ was lower. These results suggested that targeting SLC7 A11 attenuated intestinal barrier dysfunction and inflammation-related damage in mouse models with colitis.

The integrity and function of the gut barrier relies on several aspects, including innate immunity, gut microbiota, and mechanical barriers mainly composed of tight junctions [38]. These factors play a vital role in maintaining the stability and defense function of the intestinal barrier. Therefore, we validated the molecular mechanism of targeting SLC7 A11 in alleviating colitis from two directions: intestinal mechanical barrier and innate immunity of dendritic cells. TNF-α and IFN-γ were widely used as damage factors in the Caco-2 monolayer at clinically relevant concentrations [39], mimicking intestinal barrier damage associated with UC inflammation. TNF-α/IFN-γ-induced disruption of intestinal barrier function is associated with decreased expression of tight junction proteins and aberrant localization of these proteins within the cell membrane [40]. This aberrant protein localization and decreased expression is a key mechanism of impaired intestinal barrier integrity [41]. In this study, the effect of targeting SLC7 A11 on colitis-related intestinal barrier damage was investigated by incubation with a Caco-2 monolayer membrane supplemented with 1 A4 and TNF-α/IFN-γ. 1 A4 significantly reduced TNF-α/IFN-γ-induced damage, which was manifested by an increase in TEER and a decrease in FD-4 flux. We further investigated the expression and localization status of tight junction proteins. Immunofluorescence showed that 1 A4 could alleviate the localization changes of ZO-1 and Occludin induced by TNF-α/IFN-γ. Previous studies have attributed alterations in tight junction protein localization to activation of the MLCK-P-MLC signaling pathway as well as alterations in tight junctions [42]. Next, the activation of the MLCK-P-MLC signal was detected by western blotting. Targeting SLC7 A11 reduced the increase in MLCK expression and the increase in MLC phosphorylation levels.

Intestinal epithelial cells require constant renewal to maintain optimal absorption and barrier function. Apoptotic cells can lead to the development of inflammation if they are not cleared in time [38]. Amino acid transporters SLC7 A11 have been shown to be negative regulators of efferocytosis [22]. We extracted and induced BMDCs from WT and SLC7 A11^−/+^ mice bone marrow with the addition of 1 A4. 1 A4 could significantly increase the efferocytosis of WT and SLC7 A11^−/+^ BMDC. Phosphorylation levels of ERK and efferocytosis markers increased with the addition of 1 A4 and SLC7 A11^−/+^ BMDC itself. After inhibiting ERK phosphorylation, the 1 A4-enhanced efferocytosis was inhibited, indicating that the ERK pathway is a key pathway that targeting SLC7 A11 enhanced the efferocytosis of dendritic cells.

In conclusion, our findings suggest SLC7 A11 increase may be considered a molecular feature of UC. SLC7 A11 may compromise intestinal barrier integrity through the MLCK-P-MLC signaling pathway and enhance dendritic cell efferocytosis via the ERK1/2 pathway. These insights reveal novel mechanisms underlying UC pathogenesis and suggest that SLC7 A11 could serve as a promising therapeutic target for this condition.

## Supplementary Information

Below is the link to the electronic supplementary material.

Supplementary file1 (DOCX 872 KB)

## Data Availability

No datasets were generated or analysed during the current study.
