# Supplementary material for: Targeting SLC7 A11 Ameliorates Ulcerative Colitis by Promoting Efferocytosis Through the ERK1/2 Pathway
Source: Inflammation. 2025 May 13;48(6):4080–93. doi: 10.1007/s10753-025-02312-6 (PMC12722335; doi:10.1007/s10753-025-02312-6)
Supplement: Supplementary file 1 — Supplementary file1 (DOCX 872 KB) [file 10753_2025_2312_MOESM1_ESM.docx]

**Targeting SLC7A11 ameliorates ulcerative colitis by promoting efferocytosis through the ERK1/2 pathway**

Meiyi You^1^, Jichang Li^1^, Xin Wang^1^, Yucun Liu^1^, Shanwen Chen^1^, Pengyuan Wang^1^

1, Department of Gastrointestinal Surgery, Peking University First Hospital, Beijing, 100034, People’s Republic of China

Correspondence: Shanwen Chen, Department of Gastrointestinal Surgery, Peking University First Hospital, Beijing, 100034, People’s Republic of China, Email: shanwen@pku.edu.cn; Pengyuan Wang, Department of Gastrointestinal Surgery, Peking University First Hospital, Beijing, 100034, People’s Republic of China, Email: pengyuan_wang@bjmu.edu.cn


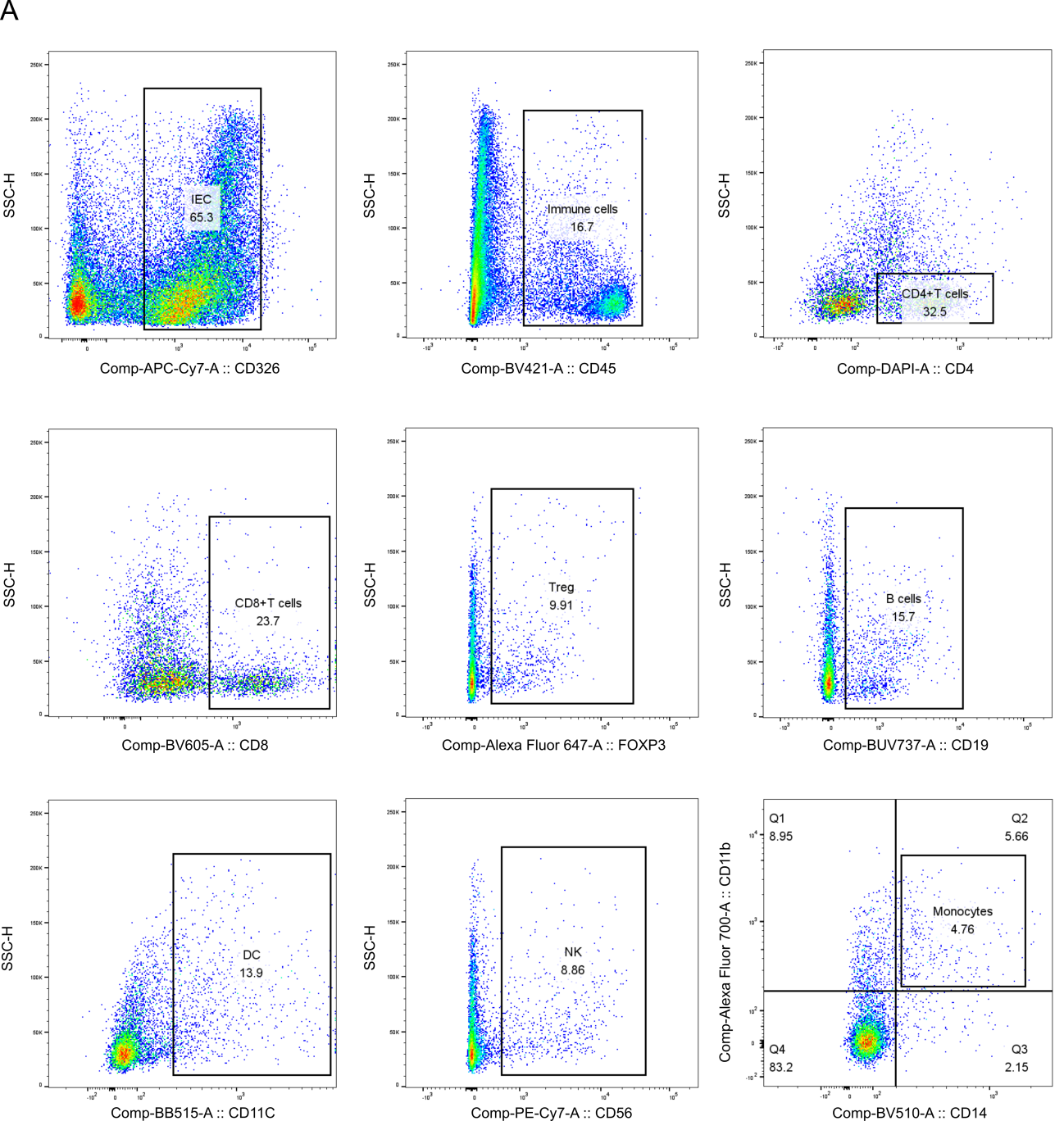


**Fig.S1**

**A** Markers of various cells in flow cytometry.

**
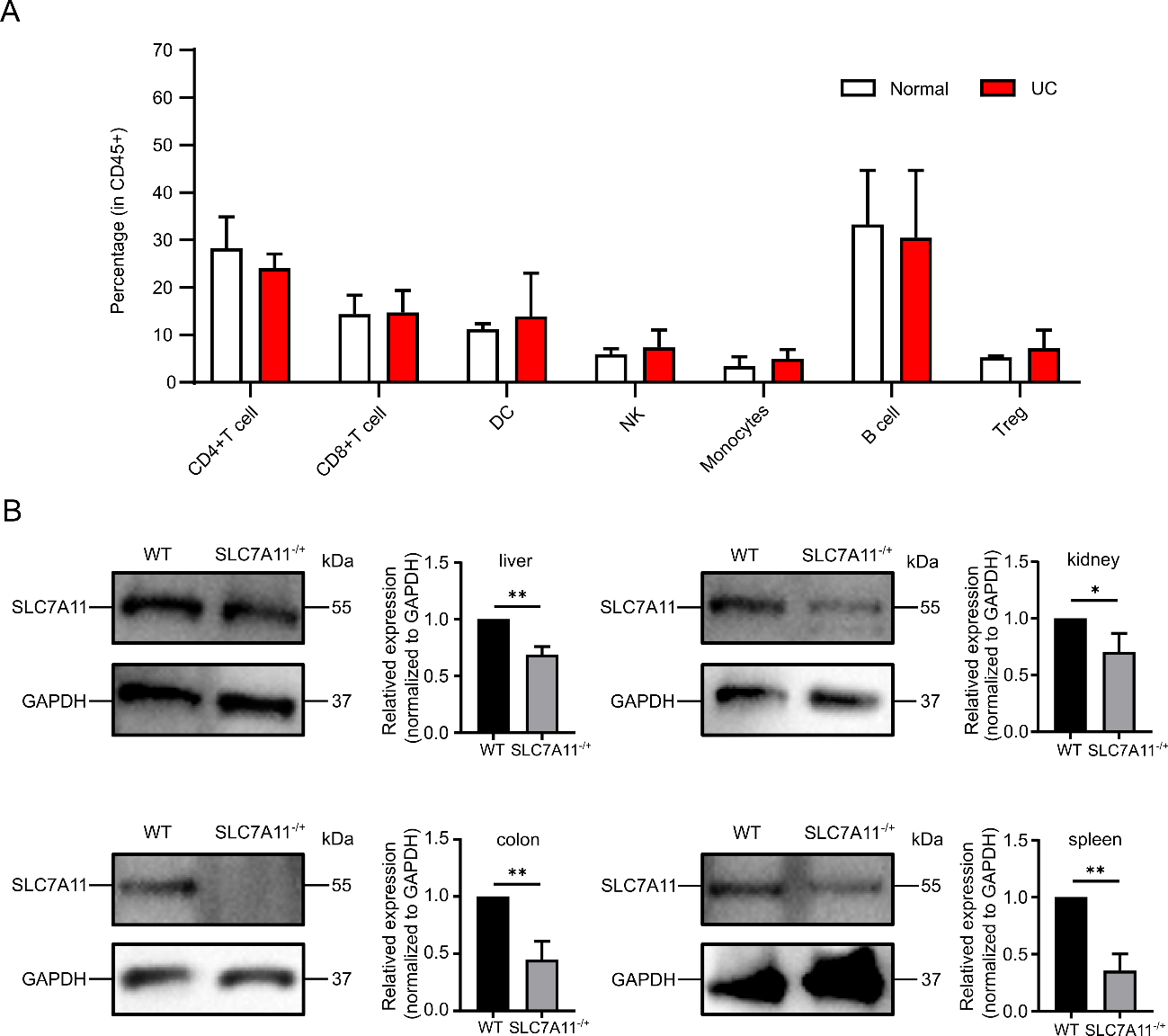
**

**Fig.S2**

**A** Proportion of individual immune cells counted by flow cytometry in inflamed colonic epithelial tissue and normal colonic epithelial tissue of UC patients. **B** Western blotting analysis of SLC7A11 protein expression in different tissues of WT and SLC7A11^-/+^ mice.
